# Supplementary material for: Microbial enzymes induce colitis by reactivating triclosan in the mouse gastrointestinal tract
Source: Nat Commun. 2022 Jan 10;13:136. doi: 10.1038/s41467-021-27762-y (PMC8748916; doi:10.1038/s41467-021-27762-y)
Supplement: Supplementary file 1 — Supplementary Information [file 41467_2021_27762_MOESM1_ESM.pdf]

Supplemental information for

Microbial Enzymes Induce Colitis by Reactivating Triclosan in the Mouse Gastrointestinal Tract

Jianan Zhang [1,*], Morgan E. Walker [2,*], Katherine Z. Sanidad [1,*], Hongna Zhang [3,4*], Yanshan Liang [3], Ermin Zhao [1], Katherine Chacon-Vargas [1], Vladimir Yeliseyev [5], Julie Parsonnet [6], Thomas D. Haggerty [6], Guangqiang Wang [1,7], Joshua B. Simpson [2], Parth B. Jariwala [2], Violet V. Beaty [2] , Jun Yang [8], Haixia Yang [1], Anand Panigrahy [1], Lisa M. Minter [9], Daeyoung Kim [10], John G. Gibbons [1], LinShu Liu [11], Zhengze Li [1], Hang Xiao [1], Valentina Borlandelli [12], Hermen S. Overkleeft [12], Erica W. Cloer [13], Michael B. Major [14], Dennis Goldfarb [15], Zongwei Cai [3,#], Matthew R. Redinbo [2,#,] and Guodong Zhang [1,16,#]

[1] Department of Food Science, University of Massachusetts, Amherst, MA, USA.
[2] Departments of Chemistry, Biochemistry, Microbiology and Genomics, University of North Carolina at Chapel Hill, Chapel Hill, NC, USA.
[3] State Key Laboratory of Environmental and Biological Analysis, Department of Chemistry, Hong Kong Baptist University, Hong Kong, SAR, China.
[4] Department of Occupational and Environmental Health, School of Public Health, Qingdao University, Qingdao, China.
[5] Massachusetts Host-Microbiota Center, Department of Pathology, Brigham and Women's Hospital, Boston, MA, USA.
[6] Department of Medicine and Department of Health Research and Policy, Stanford University, Stanford, CA, USA.
[7] School of Medical Instrument and Food Engineering, University of Shanghai for Science and Technology, Shanghai, China.
[8] Department of Entomology and Nematology, University of California, Davis, CA, USA.
[9] Department of Veterinary & Animal Sciences, University of Massachusetts, Amherst, MA, USA.
[10] Department of Mathematics and Statistics, University of Massachusetts, Amherst, MA, USA.
[11] Eastern Regional Research Center, Agricultural Research Service, United States Department of Agriculture, Wyndmoor, PA, USA.
[12] Department of Bioorganic Synthesis, Leiden Institute of Chemistry, Leiden University, Leiden, Netherlands.
[13] Lineberger Comprehensive Cancer Center, University of North Carolina at Chapel Hill, Chapel Hill, NC, USA.
[14] Department of Cell Biology and Physiology, and Department of Otolaryngology, Washington University, St. Louis, MO, USA.
[15] Department of Cell Biology and Physiology, Institute for Informatics, Washington University, St. Louis, MO, USA.
[16] Department of Food Science and Technology, National University of Singapore, Singapore.

Table S1. Concentrations of TCS, TCS-G, and TCS-Sulfate in human stool samples

Human subject #	Treatment	Treatment time (months)	TCS metabolites (pmol/ g stool)		
			TCS	TCS-G	TCS-Sulfate
2	no TCS	0	14.93	< LOD *	< LOD
5	no TCS	0	13.15	5.21	< LOD
6	no TCS	0	10.10	< LOD	< LOD
7	no TCS	0	23.67	< LOD	< LOD
9	no TCS	0	540.07	< LOD	< LOD
11	no TCS	0	19.95	< LOD	< LOD
14	no TCS	0	10.11	< LOD	< LOD
2	no TCS	1	22.49	< LOD	< LOD
5	no TCS	1	13.96	< LOD	< LOD
6	no TCS	1	18.83	< LOD	< LOD
7	no TCS	1	9.65	4.99	< LOD
9	no TCS	1	25.43	21.75	< LOD
11	no TCS	1	< LOD	< LOD	< LOD
14	no TCS	1	14.00	< LOD	< LOD
5	no TCS	2	22.69	< LOD	< LOD
6	no TCS	2	29.34	< LOD	< LOD
7	no TCS	2	19.95	< LOD	< LOD
9	no TCS	2	14.40	< LOD	< LOD
11	no TCS	2	< LOD	< LOD	< LOD
14	no TCS	2	56.73	< LOD	< LOD
2	no TCS	3	11.79	17.62	< LOD
5	no TCS	3	7.44	< LOD	< LOD
6	no TCS	3	< LOD	< LOD	< LOD
7	no TCS	3	5.90	< LOD	< LOD
9	no TCS	3	7.60	< LOD	< LOD
11	no TCS	3	7.75	< LOD	< LOD
14	no TCS	3	11.01	16.46	< LOD
2	no TCS	4	< LOD	< LOD	< LOD
5	no TCS	4	20.54	< LOD	< LOD
6	no TCS	4	< LOD	< LOD	< LOD
7	no TCS	4	8.75	< LOD	< LOD
9	no TCS	4	7.48	14.59	< LOD
11	no TCS	4	< LOD	< LOD	< LOD
14	no TCS	4	6.77	< LOD	< LOD
3	TCS	0	< LOD	10.69	0.311
4	TCS	0	12.62	5.96	< LOD
8	TCS	0	10.84	< LOD	< LOD
12	TCS	0	< LOD	< LOD	< LOD

13	TCS	0	388.08	10.80	< LOD
16	TCS	0	57.90	< LOD	< LOD
3	TCS	1	1094.80	10.24	1.344
4	TCS	1	225.14	13.69	< LOD
8	TCS	1	1049.03	< LOD	< LOD
12	TCS	1	626.33	2.81	0.342
13	TCS	1	215.36	< LOD	0.381
16	TCS	1	317.47	< LOD	< LOD
3	TCS	2	905.85	4.42	0.999
4	TCS	2	372.82	3.18	< LOD
12	TCS	2	713.18	2.97	< LOD
13	TCS	2	245.09	< LOD	< LOD
16	TCS	2	96.24	< LOD	< LOD
3	TCS	3	515.81	7.01	0.987
4	TCS	3	334.49	5.96	< LOD
8	TCS	3	515.22	< LOD	< LOD
12	TCS	3	400.99	< LOD	< LOD
13	TCS	3	445.98	6.49	< LOD
16	TCS	3	105.82	< LOD	< LOD
3	TCS	4	633.76	7.57	0.971
4	TCS	4	67.29	< LOD	< LOD
8	TCS	4	968.25	< LOD	< LOD
12	TCS	4	633.96	3.12	0.236
13	TCS	4	135.75	4.69	< LOD
16	TCS	4	295.56	< LOD	< LOD

* LOD: limit of detection.

Table S2. Concentrations of TCS, TCS-G, and TCS-Sulfate in human urine samples

Human subject #	Treatment	Treatment time (months)	TCS metabolites (nM urine)		
			TCS	TCS-G	TCS-Sulfate
5	no TCS	0	< LOD	< LOD	< LOD
9	no TCS	0	< LOD	552	0.155
16	no TCS	0	< LOD	3.52	< LOD
14	no TCS	1	< LOD	40.9	< LOD
7	no TCS	3	< LOD	2.43	< LOD
5	no TCS	4	< LOD	< LOD	< LOD
6	no TCS	4	< LOD	< LOD	< LOD
7	no TCS	4	< LOD	< LOD	< LOD
9	no TCS	4	< LOD	< LOD	< LOD
3	TCS	0	< LOD	18.4	0.071
4	TCS	0	< LOD	3.47	< LOD
8	TCS	0	< LOD	< LOD	< LOD
12	TCS	0	< LOD	12.5	< LOD
12	TCS	1	< LOD	626	0.470
12	TCS	2	8.55	953	0.522
13	TCS	3	< LOD	771	0.157
3	TCS	4	11.23	1373	0.689
4	TCS	4	5.52	775	0.388
13	TCS	4	3.06	1037	0.257
16	TCS	4	6.42	70.9	< LOD

Table S3. Crystallography statistics for two novel structures presented

	R. hominis 3 (Rh3) GUS	***F. prausnitzii* 2-L1 (Fp2-L1) GUS + GUSi-glucuronic acid**
Wavelength	1.00	1.00
Resolution range, Å (highest shell)	38 - 2.4 (2.49 - 2.4)	42 - 2.2 (2.28 - 2.2)
Space group	$P\ 2_1$	$P\ 2_1\ 2_1\ 2_1$
Unit cell (a, b, c, a, b, g; Å, deg)	54.4, 161, 86.9, 90, 107, 90	114, 128, 177, 90, 90, 90
Total reflections	190,354 (19,013)	746,484 (75,240)
Unique reflections	55,112 (5470)	132,718 (13,115)
Multiplicity	3.5 (3.5)	5.6 (5.7)
Completeness, %	98.9 (98.4)	99.9 (99.9)
Mean I/sigma(I)	18.7 (8.4)	12.7 (3.8)
Wilson B-factor, Å^2	23	23
R-merge	0.050 (0.124)	0.103 (0.447)
R-meas	0.056 (0.147)	0.114 (0.492)
R-pim	0.0319 (0.078)	0.0477 (0.204)
CC1/2	0.997 (0.981)	0.997 (0.912)
CC*	0.999 (0.995)	0.999 (0.977)
Reflections used in refinement	55,063 (5469)	132,686 (13,115)
Reflections used for R-free	2013 (202)	1999 (198)
R_{work}	0.146 (0.168)	0.150 (0.173)
R_{free}	0.187 (0.232)	0.199 (0.243)
CC_{work}	0.969 (0.946)	0.970 (0.944)
CC_{free}	0.946 (0.839)	0.950 (0.908)
Number of non-hydrogen atoms	11,018	20,834
Number of macromolecular atoms	10,112	19,055
Number of ligand atoms	76	164
Number of solvent atoms	830	1615
Number of protein residues	1268	2370
RMS_{bonds} Å	0.007	0.008
RMS_{angles} deg.	1.2	0.95
Ramachandran favored, %	97.2	96.9
Ramachandran allowed, %	2.77	2.98
Ramachandran outliers, %	0	0.04
Rotamer outliers, %	0.09	0
Clashscore	6.01	4.62
Average B-factor, all atoms, Å^2	25.2	20.7
Average B-factor, macromolecules, Å^2	24.8	20.1
Average B-factor, ligands, Å^2	30.1	35.5
Average B-factor, solvent, Å^2	29.5	26.0

Table S4. Instrumental method for the quantification of TCS, TCS-G, and TCS-Sulfate

Instrument	Thermo Scientific Dionex Ultimate 3000 UHPLC system coupled with a TSQ Quantiva Triple Quadrupole Mass Spectrometer		
Analytical column and temperature	ACQUITY UPLC C18 column (1.7 μm particles, 2.1 mm × 100 mm, Waters), 30 °C		
Mobile phases	A. Acetonitrile B. Ammonium acetate (2 mM) in water		
Gradient profile	Time (min)	Percentage A (%)	Flow rate (mL/min)
	0.0	15	0.30
	1.0	15	0.30
	2.5	80	0.30
	5.0	80	0.30
	5.5	100	0.30
	6.5	100	0.30
	7.0	15	0.30
	9.0	15	0.30
Injection volume	10 μL		
MS scan mode	Multiple reaction monitoring (MRM)		
Monitored MRM transitions	Analytes	MRM transition (*m*/*z*)	Collision Energy (eV)
	TCS	286.89 → 35.22	16
		288.89 → 35.22	16
	$^{13}C_{12}$-TCS	299.00 → 35.22	16
		301.00 → 35.22	16
	TCS-G	463.00 → 287.00	15
		465.00 → 289.00	15
	TCS-Sulfate	366.89 → 286.89	15
		368.89 → 288.89	15
MS/MS parameters	Electrospray ionization (ESI): negative ionization mode;<br>Capillary voltage (kV) = 2.5;<br>Sheath gas (arbitrary units) = 40;<br>Auxiliary gas (arbitrary units) = 10;<br>Ion transfer tube temperature (°C) = 350;<br>Vaporizer temperature (°C) = 300.		

Table S5. Site-directed mutagenesis

GUS and Residue Mutation	Forward Primer	Reverse Primer
FpL2-1 M362A	GAAGTGCCTGCTGTCGGTTTTGCG GAATCTACCATGAACT	AGTTCATGGTAGATTCCGCAAAAC CGACAGCAGGCACTTC
FpL2-1 M454A	CTACGCGGTTGTGGCGATGAGCCT GCCGAACAAC	GTTGTTCGGCAGGCTCATCGCCAC AACCGCGTAG
FpL2-1 M455A	CTACGCGGTTGTGATGGCGAGCCT GCCGAACAAC	GTTGTTCGGCAGGCTCGCCATCAC AACCGCGTAG
FpL2-1 Y479A	AACCGTTACTATGGTTGGGCCGTTA TGGGTGGCATGGG	CCCATGCCACCCATAACGGCCCAA CCATAGTAACGGTT
Rh3 F406A	CGTGATGGCGGACGTGGCCATGCT GGAAACGGAT	ATCCGTTTCCAGCATGGCCACGTC CGCCATCACG
Rh3 Y430A	CAACCTGTACTTCGGTTGGGCCATC GGTGAACTGGATCAG	CTGATCCAGTTCACCGATGGCCCA ACCGAAGTACAGGTTG
Rh3 L635STOP	CCCGGATTACATTTTCAACTAACAG GGTGACGTTG	CAACGTCACCCTGTTAGTTGAAAA TGTAATCCGGG

Table S6. **Sequences of primers**

Primers for qRT-PCR	Forward primer	Reverse primer
Gapdh	AGGTCGGTGTGAACGGATTTG	TGTAGACCATGTAGTTGAGGTCA
Tnf-α	CCCTCACACTCAGATCATCTTCT	GCTACGACGTGGGCTACAG
Il-6	TAGTCCTTCCTACCCCAATTTCC	TTGGTCCTTAGCCACTCCTTC
Mcp-1	TTAAAAACCTGGATCGGAACCAA	GCATTAGCTTCAGATTTACGGGT
Il-17	TCAGCGTGTCCAAACACTGAG	CGCCAAGGGAGTTAAAGACTT
Il-23	GGTGGCTCAGGGAAATGT	GACAGAGCAGGCAGGTACAG
Il-1β	GCAACTGTTCCTGAACTCAACT	ATCTTTTGGGGTCCGTCAACT
Il-10	GCTCTTACTGACTGGCATGAG	CGCAGCTCTAGGAGCATGTG
Tlr-4	ATGGCATGGCTTACACCACC	GAGGCCAATTTTGTCTCCACA
Ifn-γ	ATGAACGCTACACACTGCATC	CCATCCTTTTGCCAGTTCCTC
16S rRNA	CCTACGGGTGGCTGCAG	GACTACTAGGGTATCTAATCC
**Primers for sequencing**	**Forward primer (341F)**	**Reverse primer (806R)**
16S rRNA	CCTAYGGGRBGCASCAG	GGACTACNNGGGTATCTAAT

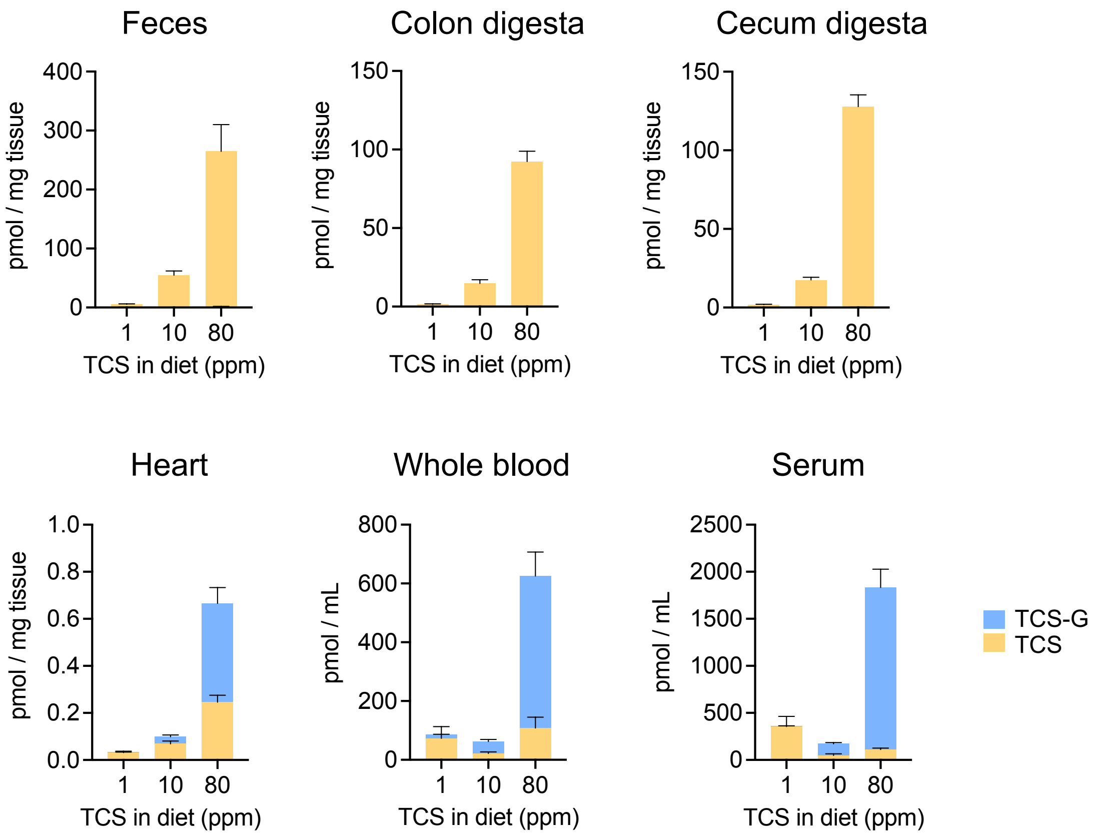

Fig. S1. TCS exposure in mice leads to accumulation of free TCS in the colon. After the mice were treated with 1, 10, and 80 ppm TCS via diet for 4 weeks, the gut tissues (feces, colon digesta, and cecum digesta) are dominated by free TCS while a mixture of TCS and its metabolite (we focused on its major metabolite TCS-G) is observed elsewhere (n = 7-8 mice per group). Part of the figure is modified from Fig. 1b. The data are mean ± SEM. Abbreviation: TCS: triclosan, TCS-G: triclosan-glucuronide.

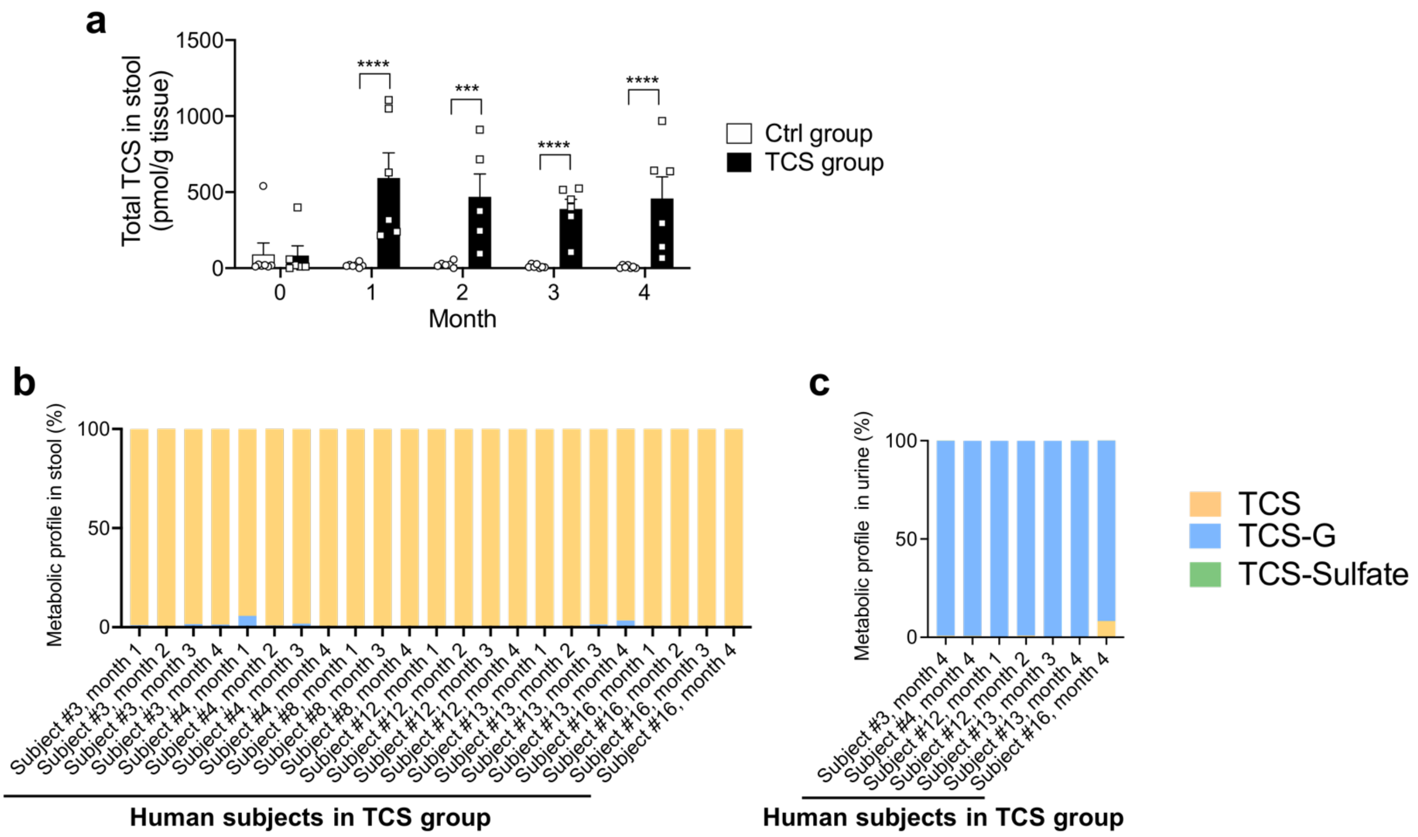

Fig. S2. TCS exposure in humans leads to accumulation of free TCS in the colon. a. Concentrations of total TCS (a combination of TCS, TCS-G, and TCS-Sulfate) in human stool samples (n=6-7). **b.** The dominant compound in the stool samples of TCS-exposed human subjects was free TCS (n = 23). The Y-axis is expressed as % metabolic profile; absolute concentrations of TCS and its metabolites are in Table S1. **c.** The dominant compound in the urine samples of TCS-exposed subjects was TCS-G (n = 7). See absolute concentrations of TCS and its metabolites in urine samples in Table S2. The data are mean ± SEM, ****P* < 0.001, *****P* < 0.0001.

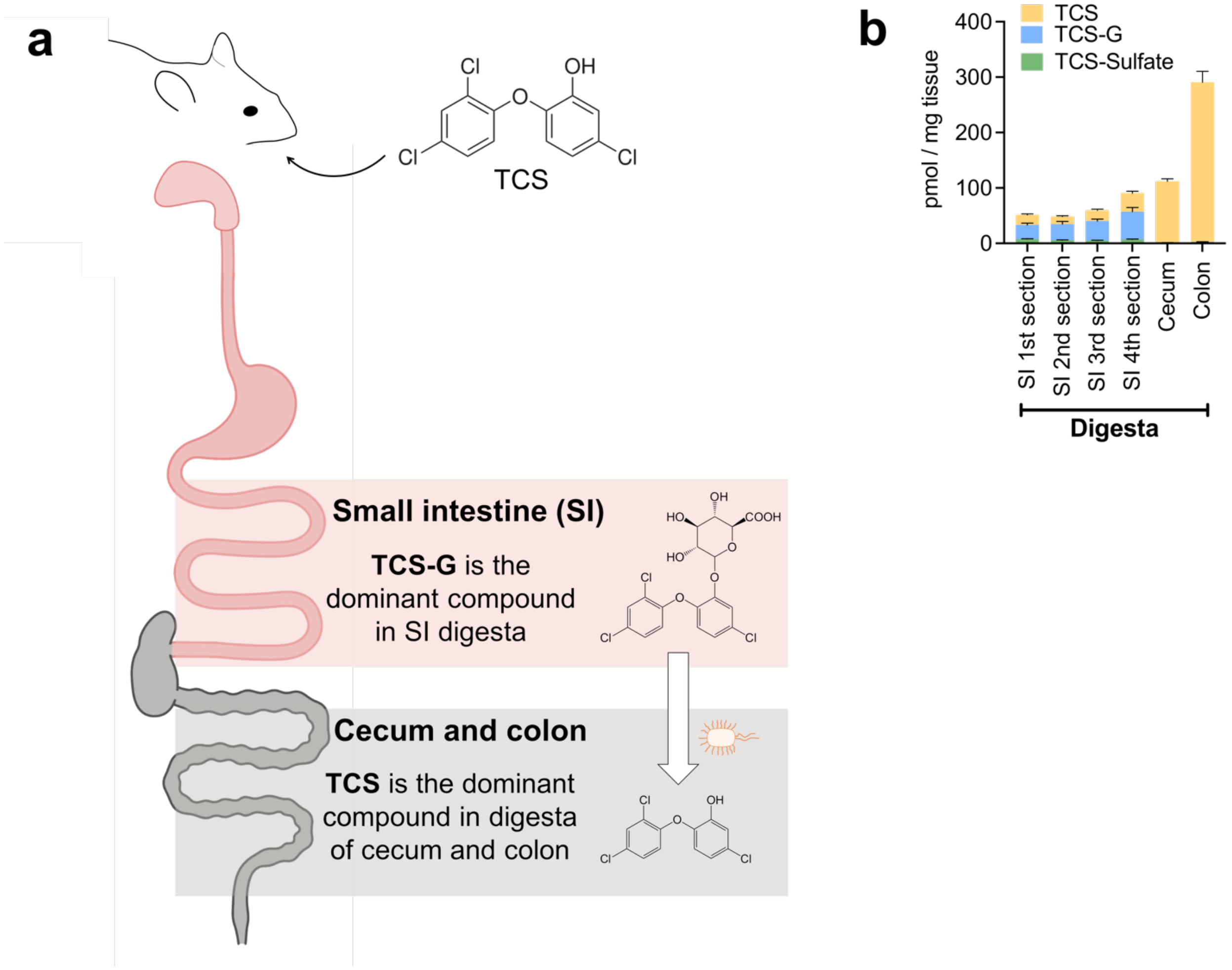

Fig. S3. Schematic of Core Hypothesis. a. Hypothesis: gut microbiota participates in the conversion of triclosan-glucuronide (TCS-G), which is derived from host metabolism, to triclosan (TCS), contributing to the accumulation of free TCS in the lower gastrointestinal tract. **b.** In support of this hypothesis, after the mice were exposed to 80 ppm TCS via diet, TCS-G was the dominant compound and free TCS was a minor compound in small intestinal digesta, while free TCS was the most abundant compound in the digesta of cecum and colon (modified from Fig. 1a, n = 10 mice per group). Part of the picture was created with BioRender.com. The data are mean ± SEM.

.

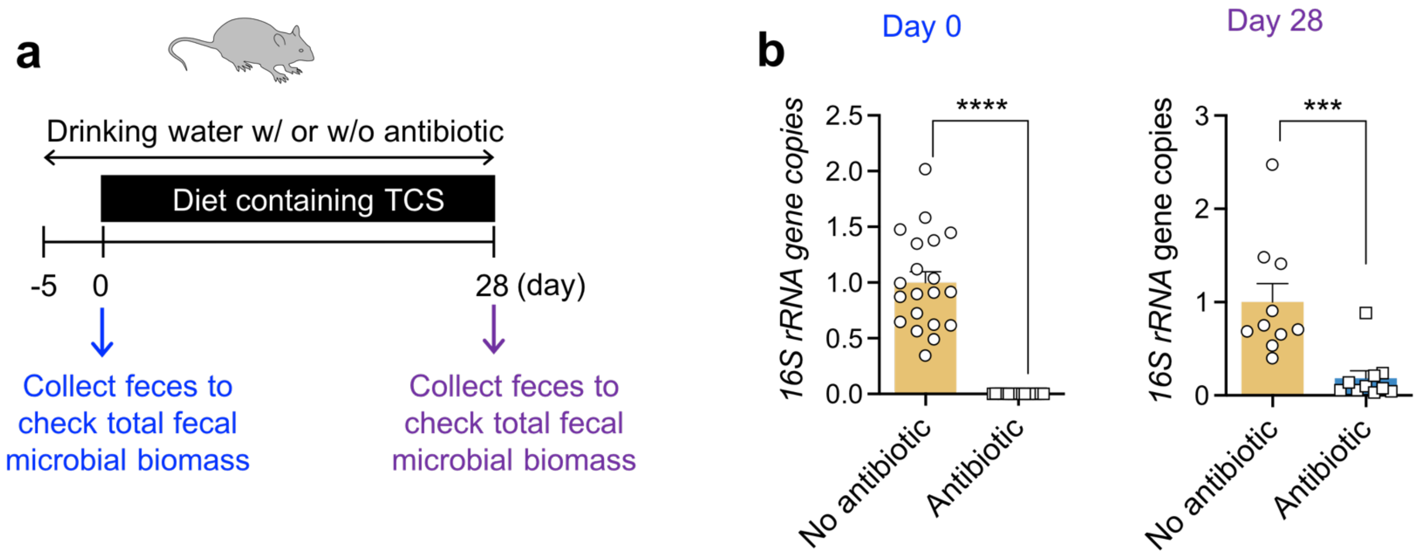

Fig. S4. Antibiotic cocktail reduces gut microbial abundance in mice. a. Mouse feces were collected on day 0 and day 28 and were analyzed using *16S rRNA* gene as a proxy marker for microbial abundance. **b.** *16S rRNA* gene copies in mouse feces showed that antibiotic cocktail treatment caused a significant reduction in fecal microbial biomass on day 0 (n = 20 mice per group) and day 28 (n = 10 mice per group). The data are mean ± SEM, ***$P < 0.001$, ****$P < 0.0001$.

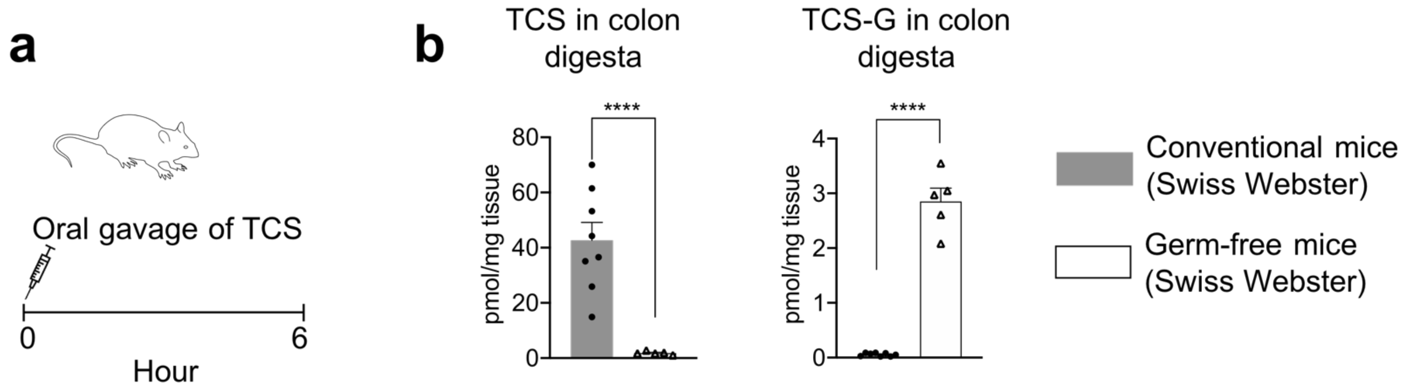

Fig. S5. TCS is reduced and TCS-G is increased in the colon digesta of germ-free Swiss Webster mice relative to conventional Swiss Webster mice. a. Conventional or germ-free Swiss Webster mice were treated with a one-time oral gavage of 8 mg/kg TCS; after 6 h, the mice were sacrificed and the levels of TCS and TCS-G in colon digesta were analyzed by LC-MS/MS. **b.** Compared with conventional animals, germ-free Swiss Webster mice had reduced TCS and increased TCS-G in colon digesta. n = 8 mice per group for conventional mice, and n = 5 mice per group for germ-free mice. The data are mean ± SEM, ****$P < 0.0001$.

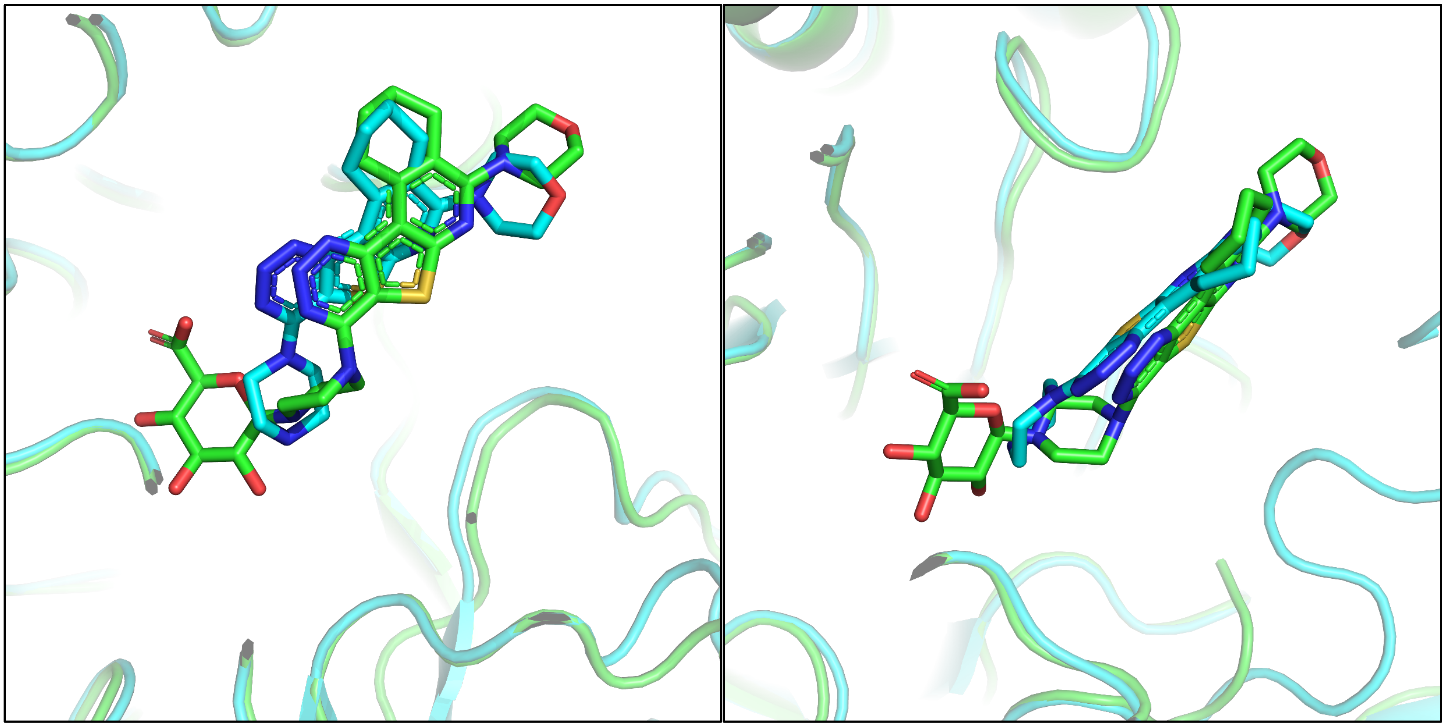

Fig. S6. **Comparison of GUSi binding modes between Fp2-L1 GUS and *C. perfringens* GUS (PDB 6CXS).** *C. perfringens* GUS (cyan) was crystallized in the presence of GUSi alone and reveals the ligand bound at the enzyme's active site. Fp2-L1 GUS (green) was crystallized in the presence of GUSi and *p*-nitrophenyl-glucuronide and reveals a covalent GUSi-glucuronic acid conjugate bound at the enyzme's active site that occupies a position akin to that adopted by GUSi alone.

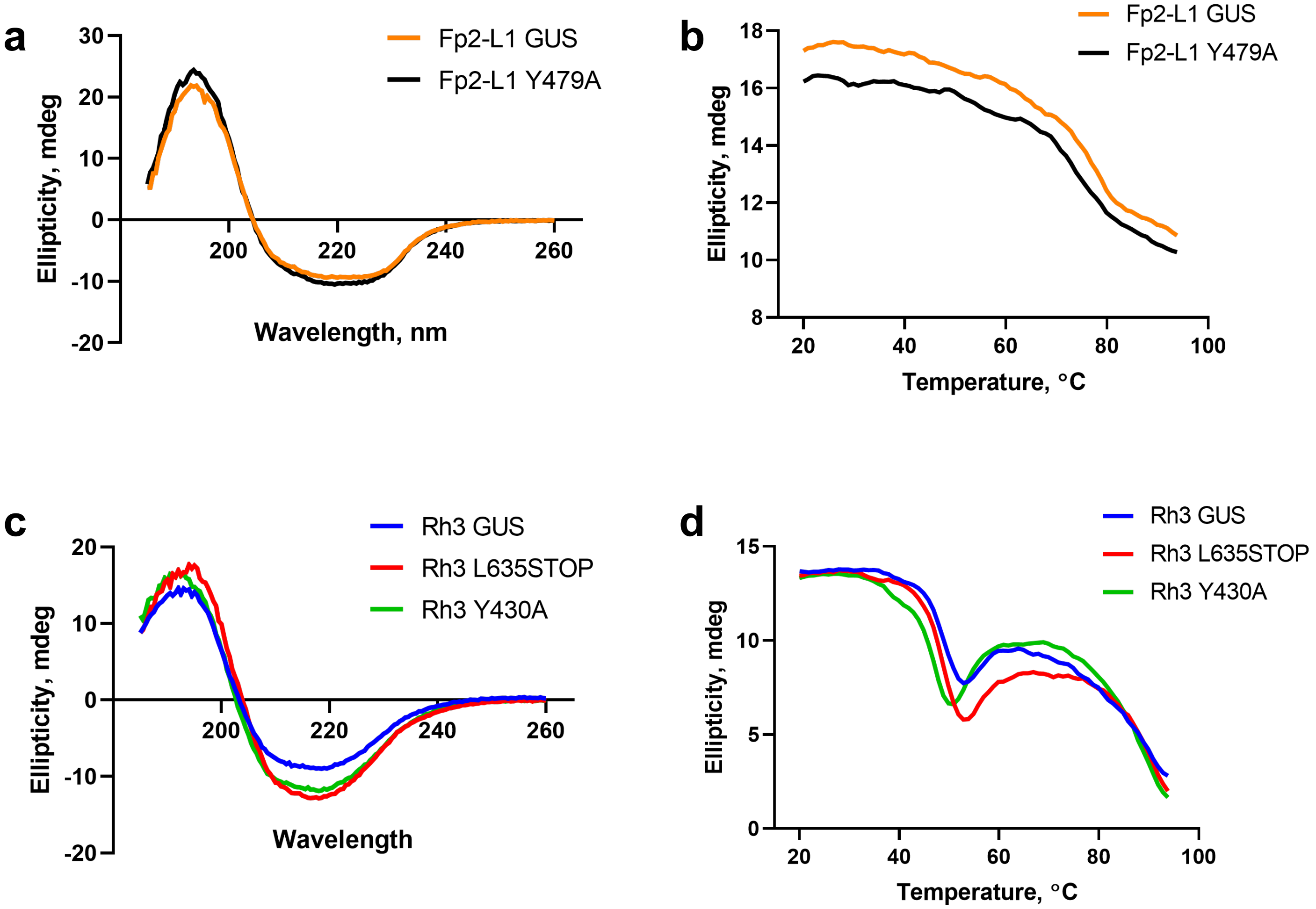

Fig. S7. **Circular dichroism analysis of wild-type and mutant GUS proteins. a.** Circular dichroism scan at 20°C of Fp2-L1 GUS wild-type and the Y479A mutant that showed no TCS-G processing activity. **b.** Circular dichroism melting profile from 20 to 94°C at 193 nm of Fp2-L1 GUS wild-type and the Y479A mutant that showed no TCS-G processing activity. **c.** Circular dichroism scan of Rh3 GUS wild-type and the mutants with no activity at 20°C. **d.** Circular dichroism melting profile of Rh3 GUS wild-type and the mutants with no activity from 20 to 94°C at 193 nm.

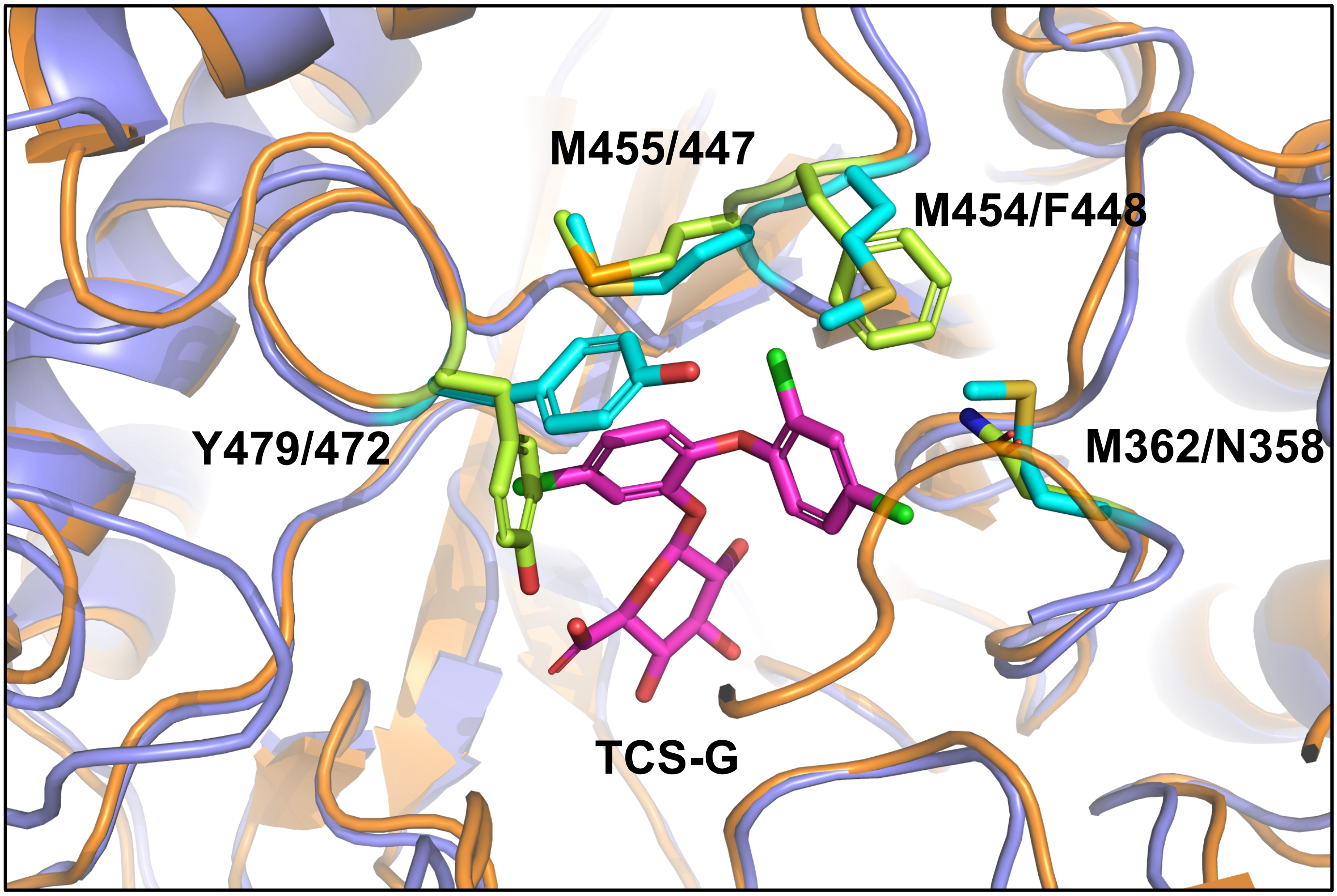

Fig. S8. Active site of Fp2-L1 GUS (purple) with TCS-G (magenta) docked superimposed on the active site of *E. coli* GUS (orange, PDB 3LPF). *E. coli* GUS, which is also a Loop 1 GUS but a poor processor of TCS-G, exhibits distinct residues (A358, F448; lime) at positions equivalent to residues in Fp2-L1 GUS shown to be critical for TCS-G processing (M362, M455; cyan). Residue labels are "Fp2-L1 GUS / *E. coli* GUS".

LOOP 1 GUS ALIGNMENT - LOOP 1 REGION

```
Lactobacillus_rhamnosus                  361    GFKMAAAAFLGG-----LNQSFFKG-PW  382
Ruminococcus_gnavus                      361    GMMRSTRNFVAAGSG--NYTYFFEA-LT  385
Faecalibacterium_prausnitzii_2-Loop      360    GFMESTMNFLAANQGNGKKVGWFEK-ET  386
Faecalibacterium_prausnitzii             359    GFMQSTANFLAANQGNGRQQGFFEK-ET  385
Escherichia_coli                         356    GFNLSLGIGFEA--GN-KPKELYSEEAV  380
Streptococcus_agalactiae                 355    GLFQNFNASLDL--SP----KDNGT-WN  375
Eubacterium_eligens                      369    GVNLQFGGGANF--GG-ERIGTFDK-EH  392
Clostridium_perfringens                  358    GLHLNFMAT-GF--GG-DAP-KRDT-WK  379
                                                *.
```

Percent Identity Matrix – LOOP 1 REGION

1: Lactobacillus_rhamnosus	100.00	18.18	22.73	36.36	15.79	26.32	18.75	11.76
2: Ruminococcus_gnavus	18.18	100.00	52.00	56.00	4.55	13.64	21.05	30.00
3: Faecalibacterium_prausnitzii_2-Loop	22.73	52.00	100.00	77.78	12.50	29.17	28.57	27.27
4: Faecalibacterium_prausnitzii	36.36	56.00	77.78	100.00	8.33	29.17	23.81	27.27
5: Escherichia_coli	15.79	4.55	12.50	8.33	100.00	25.00	9.52	22.73
6: Eubacterium_eligens	26.32	13.64	29.17	29.17	25.00	100.00	19.05	22.73
7: Streptococcus_agalactiae	18.75	21.05	28.57	23.81	9.52	19.05	100.00	28.57
8: Clostridium_perfringens	11.76	30.00	27.27	27.27	22.73	22.73	28.57	100.00

Fig. S9. Alignment and percent identity matrix of the Loop 1 region from Loop 1 GUS enzymes. Alignments were performed using the ClustalOmega Multiple Sequence Alignment tool.

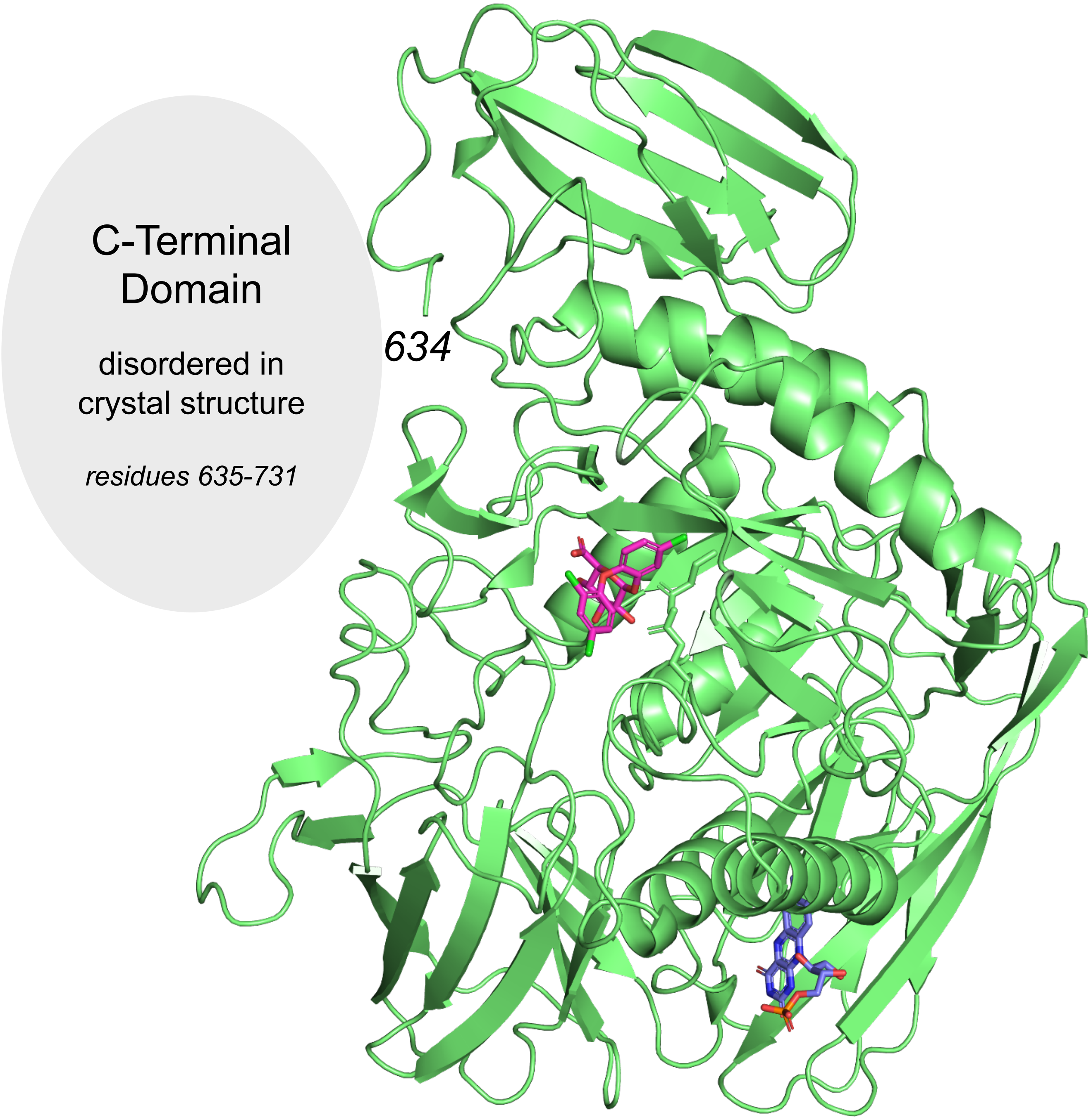

Fig. S10. The unresolved C-terminal domain of Rh3 GUS (residues 635-731) would be expected to occupy the space indicated adjacent to the Rh3 GUS monomer crystal structure (green) with docked TCS-G in magenta and bound FMN (blue).

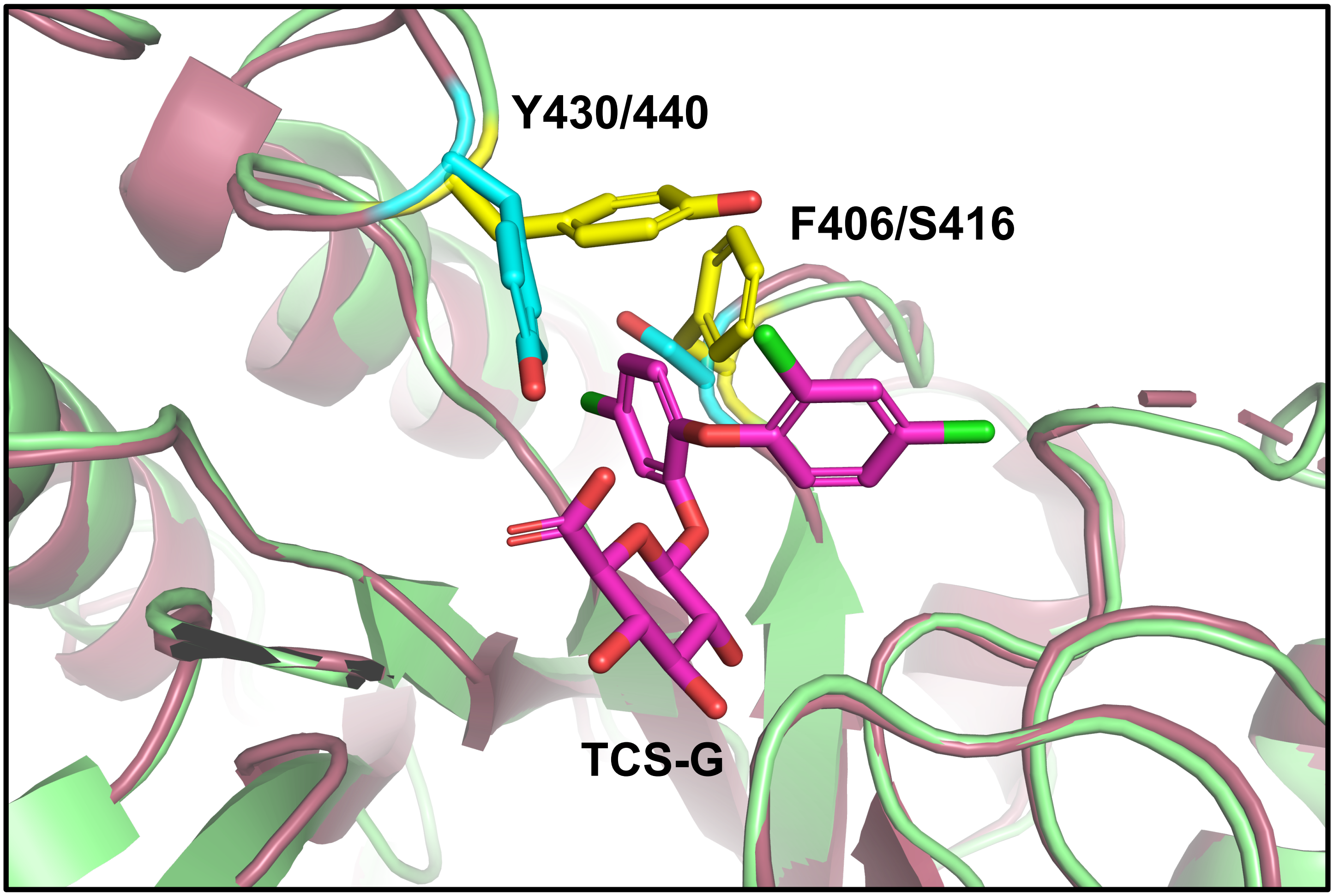

Fig. S11. Active site of Rh3 GUS (green) with TCS-G (magenta) docked superimposed on the active site of Rh2 GUS (maroon, PDB 6MVH). Rh2 GUS, which is also an FMN-binder but a poor processor of TCS-G, contains S416 (cyan) in place of F406 (yellow) in Rh3, a residue that has been shown to be critical for TCS-G processing. Residue labels are “Rh3 GUS / Rh2 GUS”.

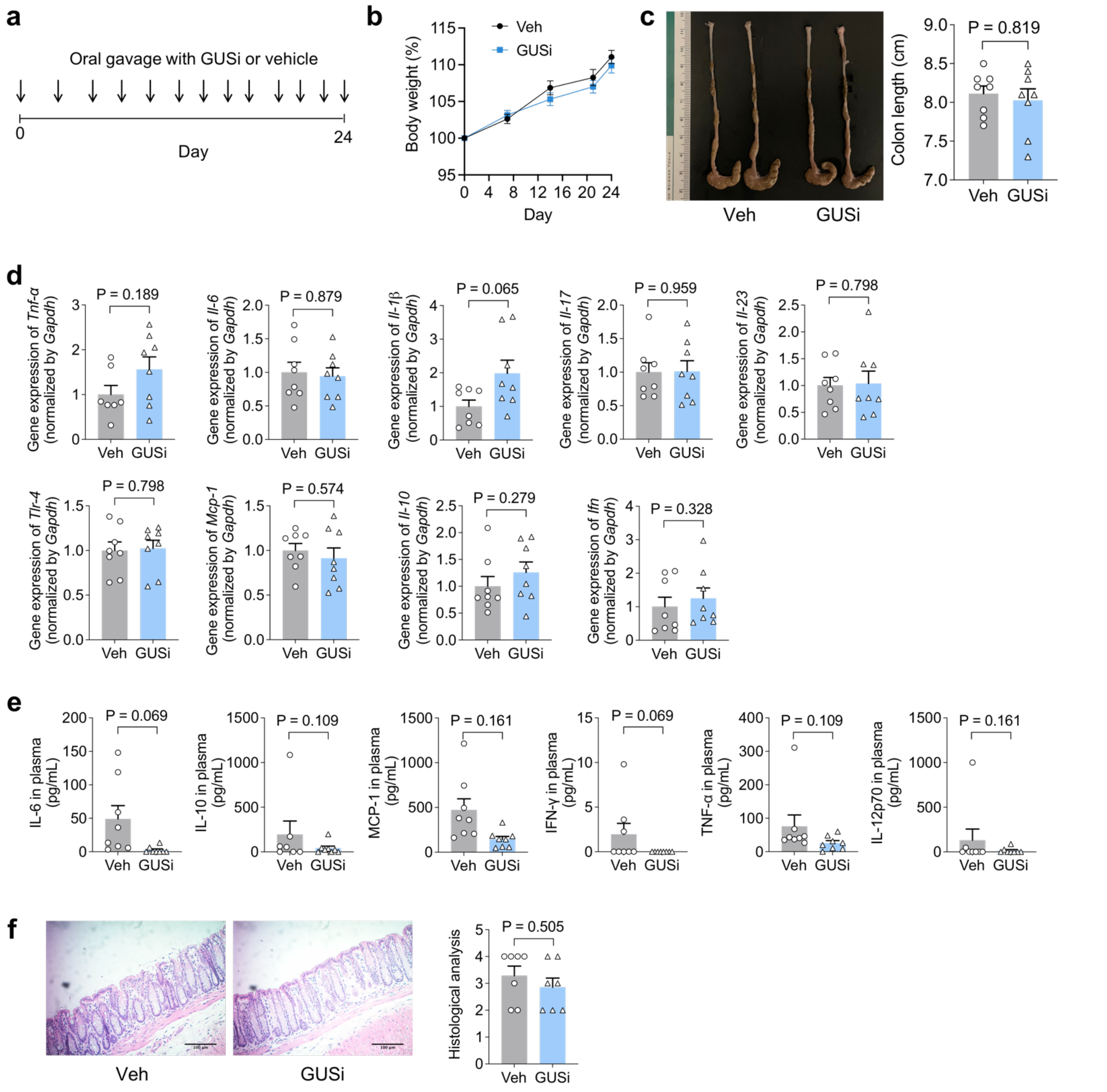

Fig. S12. GUSi treatment has little effect on colonic inflammation in mice. **a.** C57BL/6 mice were treated with 1 mg/kg GUSi or vehicle by oral gavage. At end of the experiment, the mice were sacrificed and subjected to biochemical analysis. **b.** body weight. **c.** colon length. **d.** qRT-PCR analysis of inflammatory gene expression in colon. **e.** ELISA measurement of cytokines in plasma. **f.** histology of colon tissues (scar bar =100 μm). The data are mean ± SEM, n = 8 mice per group.

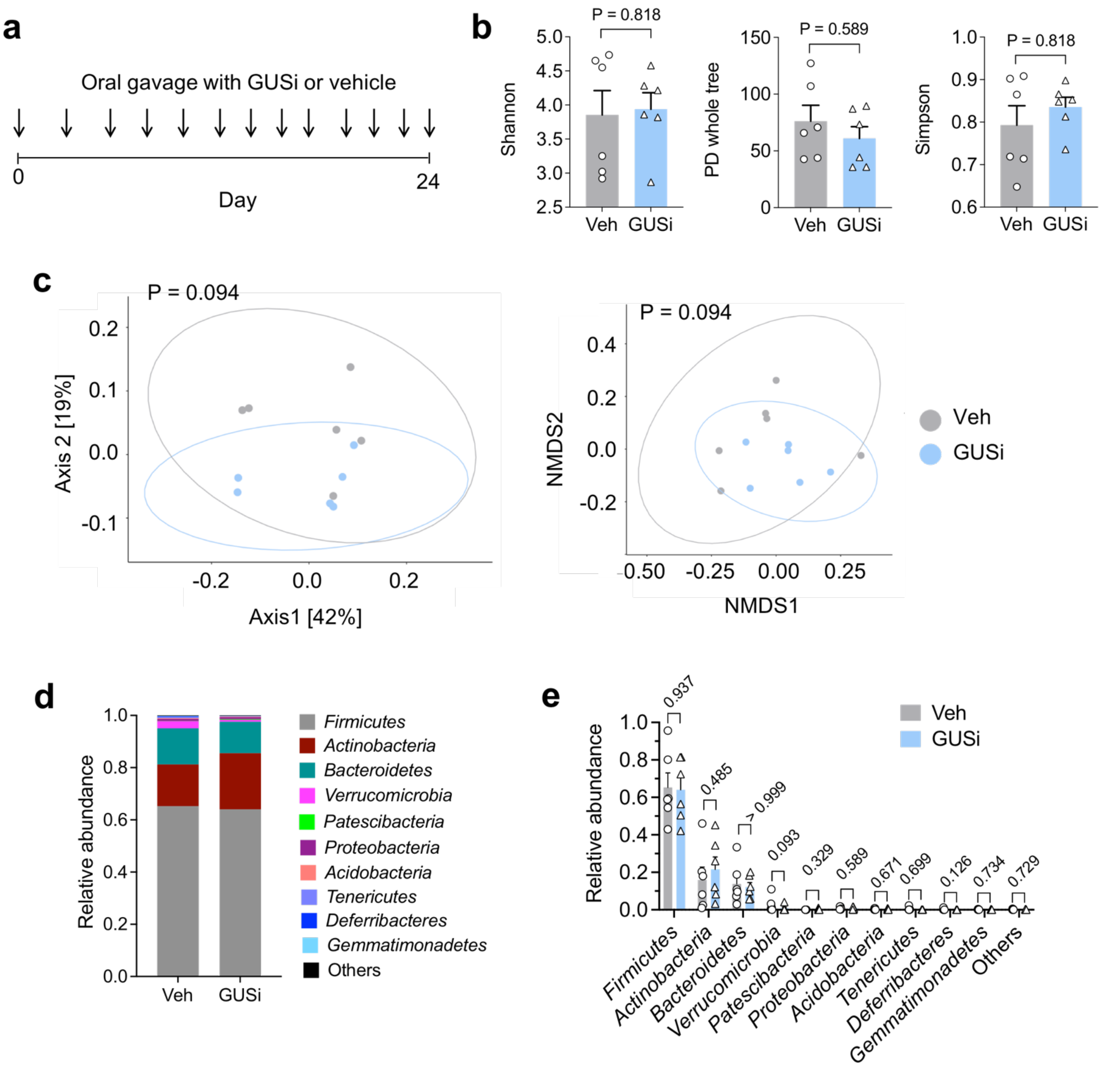

Fig. S13. GUSi treatment has little effect on the diversity or composition of gut microbiota in mice. a. C57BL/6 mice were treated with 1 mg/kg GUSi or vehicle by oral gavage. At end of the experiment, fecal material was collected and subjected to *16S rRNA* sequencing. **b.** GUSi has little effect on alpha diversity of the microbiota, as assessed using multiple diversity parameters including Shannon, PD whole tree, and Simpson. **c.** GUSi has little effect on beta diversity of the microbiota, as assessed using weighted UniFrac distance followed by Principal Coordinate Analysis (PCoA) and non-metric multidimensional scaling (NMDS) analysis. **d-e.** GUSi has little effect on composition of the microbiota at phylum levels. The data are mean ± SEM, n = 6 mice per group.

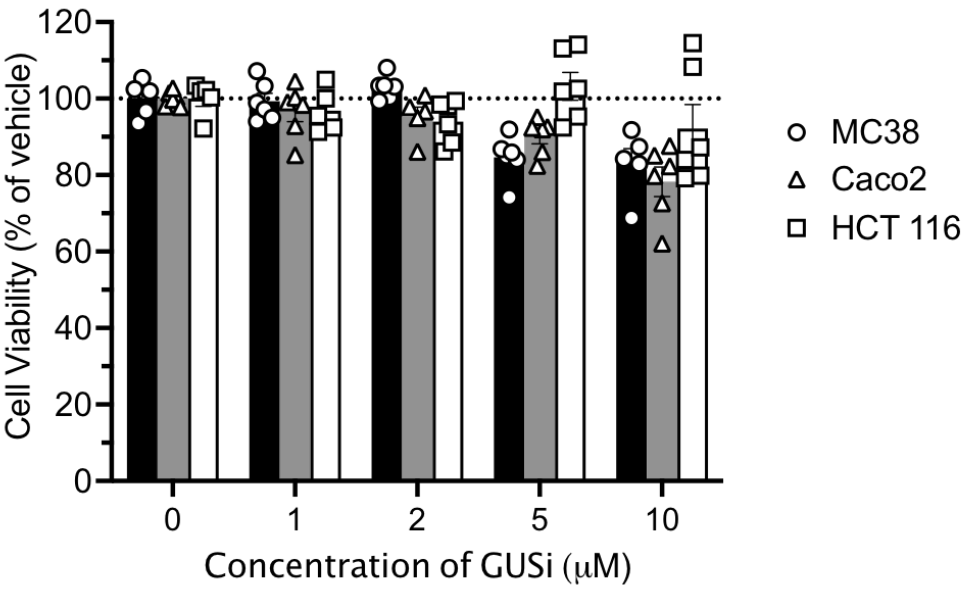

Fig. S14. GUSi treatment has little effect on the proliferation of mouse or human intestinal cells *in vitro*. Mouse (MC38) or human (Caco2 and HCT-116) intestinal cells were treated with GUSi or vehicle (0.2% v/v DMSO) for 24 h, then cell viability was analyzed using a MTT assay. The data are mean ± SEM, n = 5-6 per group.

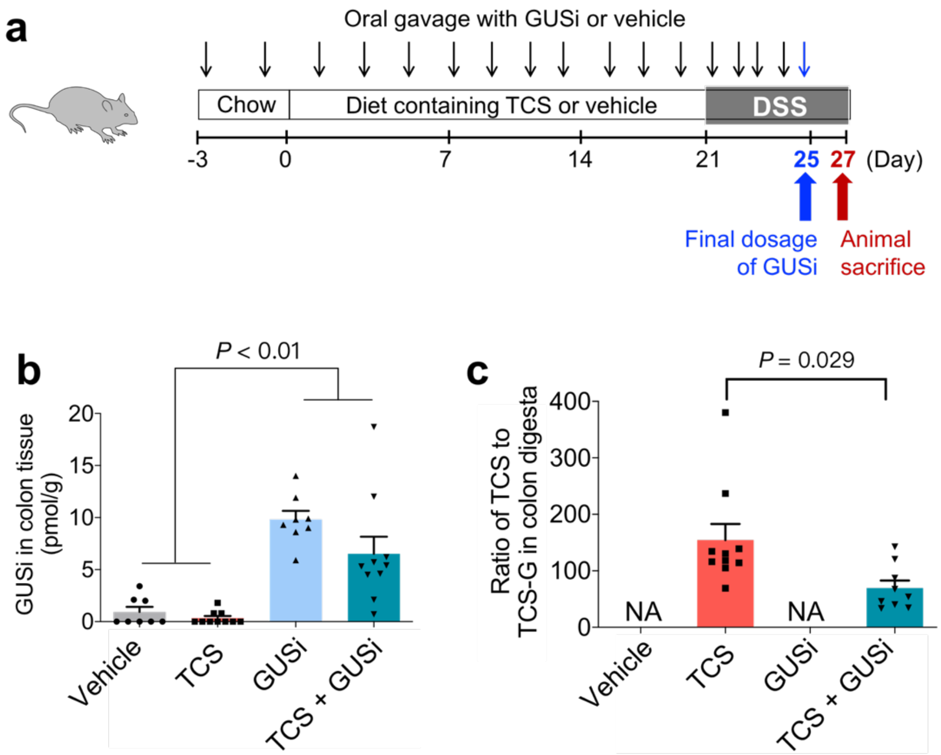

Fig. S15. Orally dosed GUSi reaches the mouse colon and suppresses conversion of TCS-G to TCS. a. C57BL/6 mice were treated with TCS or vehicle via diet, with or without co-administration of GUSi, and then mice were stimulated with DSS to induce colitis. The final GUSi was dosed on day 25 and mice were sacrificed for analysis on day 27. **b.** GUSi is detected in the mouse colon 2 days after the final dose of the inhibitor. **c.** GUSi treatment reduces the ratio of TCS to TCS-G in colon digesta. The data are mean ± SEM, n = 6-10 mice per group. NA: no TCS or TCS-G detected.

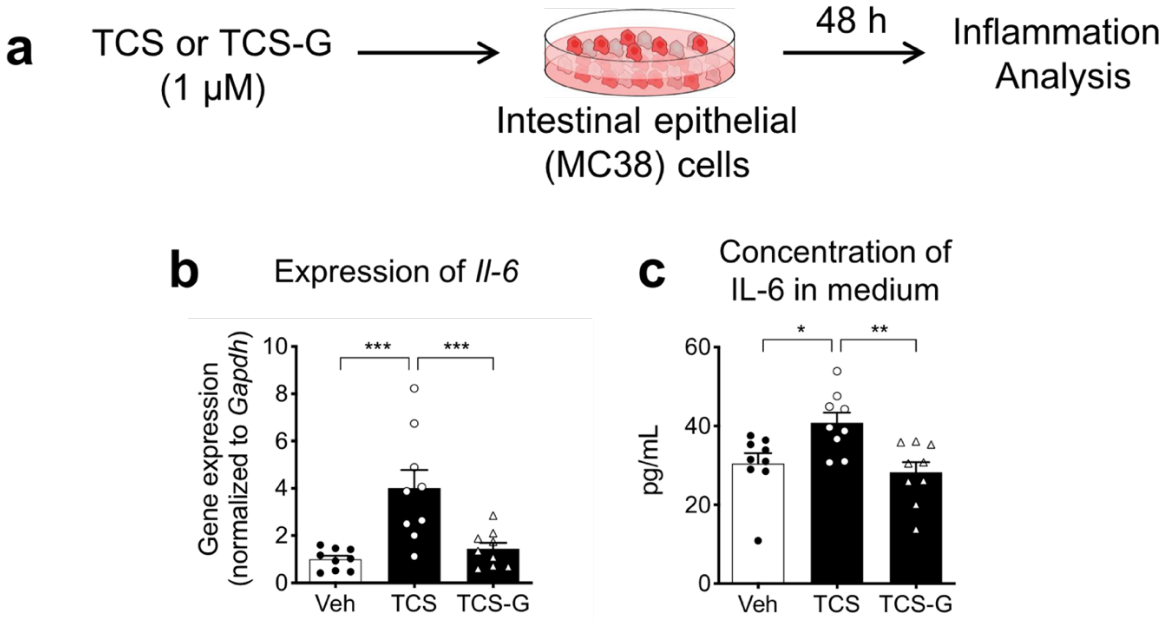

Fig. S16. TCS induces inflammatory responses in mouse intestinal epithelial cells but TCS-G does not. **a.** Cultured murine intestinal epithelial MC38 cells were treated with 1 μM of TCS or TCS-G for 48 h prior to studying inflammatory responses. **b.** TCS increases the expression of *Il-6*, which encodes the pro-inflammatory cytokine interleukin 6 (IL-6), in MC38 cells but TCS-G does not. **c.** ELISA analysis shows that TCS increases the concentration of IL-6 in cell culture medium of MC38 cells but TCS-G does not. The data are mean ± SEM, n = 3 per group from three independent experiments, * $P < 0.05$, ** $P < 0.01$, *** $P < 0.001$.

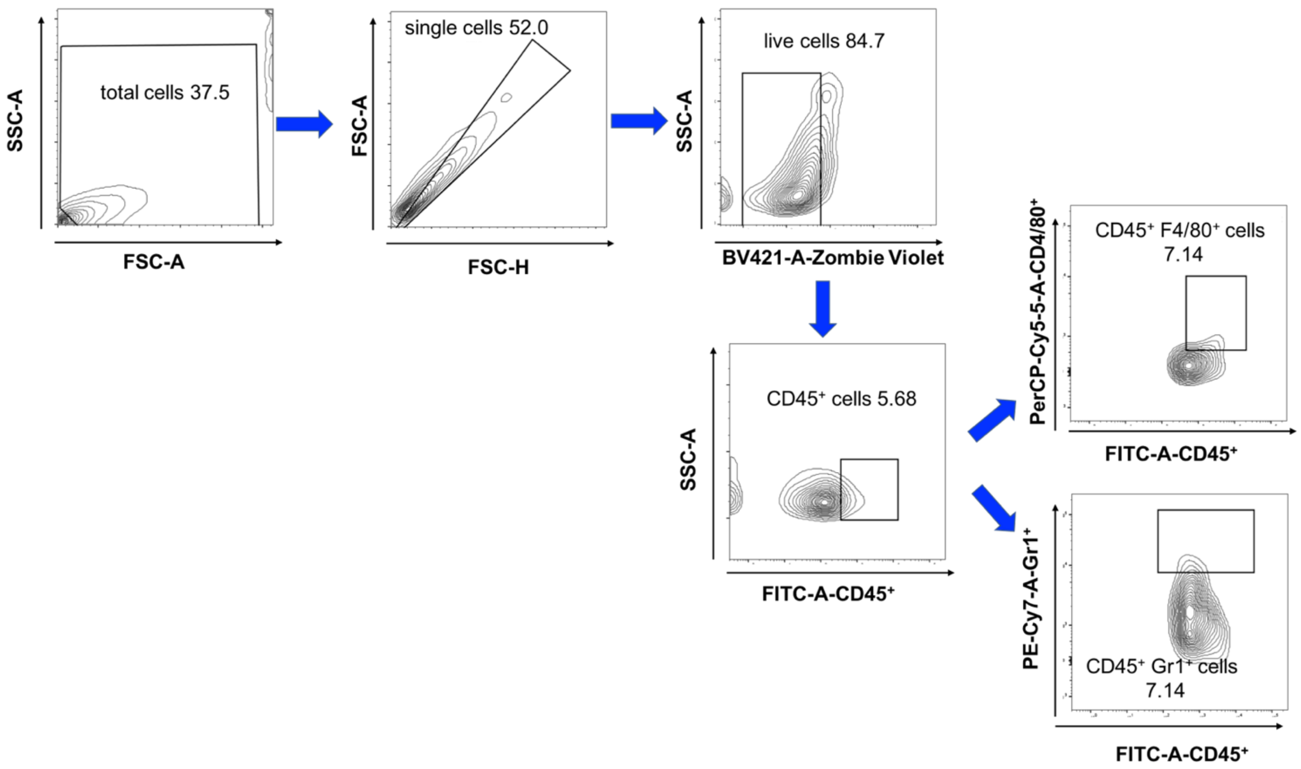

Fig. S17. Gating strategies used for the identification of major immune cell populations. The gating panel corresponds to the FACS data in Fig. 5d.
