# Supplementary material for: Microbial enzymes induce colitis by reactivating triclosan in the mouse gastrointestinal tract
Source: Nat Commun. 2022 Jan 10;13:136. doi: 10.1038/s41467-021-27762-y (PMC8748916; doi:10.1038/s41467-021-27762-y)
Supplement: Supplementary file 3 — Reporting Summary [file 41467_2021_27762_MOESM3_ESM.pdf]

nature research

Corresponding author(s): Guodong Zhang; Matthew R. Redinbo; Zongwei Cai

Last updated by author(s): Nov 7, 2021

Reporting Summary

Nature Research wishes to improve the reproducibility of the work that we publish. This form provides structure for consistency and transparency in reporting. For further information on Nature Research policies, see our Editorial Policies and the Editorial Policy Checklist.

Statistics

For all statistical analyses, confirm that the following items are present in the figure legend, table legend, main text, or Methods section.

n/a	Confirmed	
☐	☒	The exact sample size (n) for each experimental group/condition, given as a discrete number and unit of measurement
☐	☒	A statement on whether measurements were taken from distinct samples or whether the same sample was measured repeatedly
☐	☒	The statistical test(s) used AND whether they are one- or two-sided<br>*Only common tests should be described solely by name; describe more complex techniques in the Methods section.*
☐	☒	A description of all covariates tested
☐	☒	A description of any assumptions or corrections, such as tests of normality and adjustment for multiple comparisons
☐	☒	A full description of the statistical parameters including central tendency (e.g. means) or other basic estimates (e.g. regression coefficient) AND variation (e.g. standard deviation) or associated estimates of uncertainty (e.g. confidence intervals)
☐	☒	For null hypothesis testing, the test statistic (e.g. F, t, r) with confidence intervals, effect sizes, degrees of freedom and P value noted<br>*Give P values as exact values whenever suitable.*
☒	☐	For Bayesian analysis, information on the choice of priors and Markov chain Monte Carlo settings
☒	☐	For hierarchical and complex designs, identification of the appropriate level for tests and full reporting of outcomes
☒	☐	Estimates of effect sizes (e.g. Cohen's d, Pearson's r), indicating how they were calculated

Our web collection on statistics for biologists contains articles on many of the points above.

Software and code

Policy information about availability of computer code

Data collection	The LC-MS/MS data, which was acquired using a Thermo Scientific Dionex Ultimate 3000 ultrahigh performance liquid chromatography (UHPLC) system coupled with a TSQ Quantiva Triple Quadrupole Mass Spectrometer, were analyzed using Xcalibur software (version 4.1, Thermo Fisher Scientific). TCS-G docking into various GUS enzymes was carried out using the Schrödinger (http://www.schrodinger.com) induced fit docking pipeline (Release 2020-1). Mass spectrometry/proteomics data were analyzed using Metalab (version 1.1.1) with MaxQuant (version 1.6.2.3). Phenix (version 1.17.1-3660) was used to solve crystal structures via molecular replacement. Coot (version 0.9.4) was used for manual, visual inspection of crystal structures. Flow cytometry data were acquired on a BD LSR FortessaTMcell analyzer (Becton Dickinson, Franklin Lakes, NJ) and analyzed using FlowJo software (FlowJo, LLC).
Data analysis	For the comparison between two groups, Shapiro-Wilk test was used to verify the normality of data; when data were normally distributed, statistical significance was determined using two-side t-test; otherwise, significance was determined by Wilcoxon-Mann-Whitney test. The statistical comparison of three groups was analyzed using one-way ANOVA by Tukey's multiple comparison. The statistical analyses were performed using SAS (version 9.3) statistical software and GraphPad Prism (version 8.0 or 9.0). $P < 0.05$ was considered statistically significant.

For manuscripts utilizing custom algorithms or software that are central to the research but not yet described in published literature, software must be made available to editors and reviewers. We strongly encourage code deposition in a community repository (e.g. GitHub). See the Nature Research guidelines for submitting code & software for further information.

Data

Policy information about availability of data

All manuscripts must include a data availability statement. This statement should provide the following information, where applicable:

- Accession codes, unique identifiers, or web links for publicly available datasets
- A list of figures that have associated raw data
- A description of any restrictions on data availability

All data generated or analysed during this study are included in this published article (and its supplementary information files). The mass spectrometry proteomics data have been deposited to the ProteomeXchange Consortium via the PRIDE partner repository with the dataset identifier PXD025887. Final PDB coordinates were deposited to the RCSB Protein Data Bank under the codes 7KGZ [https://doi.org/10.2210/pdb7KGZ/pdb] (Rh3) and 7KGY [https://doi.org/10.2210/pdb7KGY/pdb] (FpL2-1 with GUSi).

Field-specific reporting

Please select the one below that is the best fit for your research. If you are not sure, read the appropriate sections before making your selection.

☒ Life sciences ☐ Behavioural & social sciences ☐ Ecological, evolutionary & environmental sciences

For a reference copy of the document with all sections, see nature.com/documents/nr-reporting-summary-flat.pdf

Life sciences study design

All studies must disclose on these points even when the disclosure is negative.

Sample size	The sample sizes of the animal experiments were determined based on power calculation and our previous research using similar techniques (PMID: 33338815, 31868902, 31760871, 29848663, 31444155, 30503401). The sample sizes for the cell culture studies were determined based on our previous research (PMID: 30803995). The sample sizes for the biochemical studies were determined based on our previous research using similar techniques (PMID: 32170007, 31774274, 31663730, 30729778, 30658055, 30696850).
Data exclusions	For LC-MS/MS analysis of TCS metabolism in mice and humans (Fig. 1-2) or in vitro or enzymatic assays (Fig. 3-4), we did not exclude data in figure preparation and data analysis. For the RT-PCT analysis of gene expression in colon tissues (Fig. 5E), a limited number of data sets were clearly abnormal, which was further validated by the ROUT method (a method developed to identify outliers from nonlinear regression) using Prism 9 software, then these outliers were removed from data analysis. In addition, we reported raw data either in supplemental table (Table S1-2) or as scatter-plot points in figures when feasible (such as Fig. 1, 2, and 5).
Replication	The in vitro experiments (Fig. 2A) and enzymatic assays (Fig. 3-4) were replicated three times with similar and successful results. The animal experiments (Fig. 1, 2, and 5) were performed once with enough animal size per group. Prior to the animal experiment, we determined the animal size based on power calculation and our previous research using similar techniques (PMID: 33338815, 31868902, 31760871, 29848663, 31444155, 30503401); and the animal experiments have at least 5 replicates per group in the animal study. To increase the rigor of our research, we have used multiple complementary techniques, to aid in avoiding weakness associated with each particular technique. For example, to study TCS metabolism (Fig. 1), we performed LC-MS/MS analyses in both mice and humans. To study the roles of gut microbiota involved (Fig. 2), we used a combination of in vitro culturing studies of fecal bacteria, antibiotic-mediated suppression of gut bacteria in vivo (long-term treatment and short-term time-course study), germ-free mouse models in both C57BL/6 and Swiss Webster background. To study the specific GUS enzymes involved in TCS metabolism (Fig. 3), we used a combination of enzymatic assays against a panel of purified gut microbial GUS enzymes, activity-based probe-enabled proteomics using human fecal samples, and X-ray crystallography.
Randomization	After acclimation, the mice were housed in standard cages, then randomly assigned to different experimental treatment groups. Each cage has 2 mice per cage, in order to increase the number of mouse cages and minimize the potential cage effect on gut microbiota. Randomization is not relevant to the in vitro assays performed.
Blinding	For LC-MS/MS analyses of TCS metabolism in mice and humans (Fig. 1 and Fig. 2), the sample and human samples were given a code before the experiments and this code was not available to the LC-MS/MS experimenter until data analysis. In addition, the animal experiment was performed in the UMass-Amherst campus in United States; then the animal samples were shipped to the HKBU campus in Hong Kong for LC-MS/MS analysis. Thus, the animal sample preparation and sample analysis were performed by independent investigators, which helps to reduce or eliminate experimental bias. For histological analysis of colon tissues (Fig. 5), the histologic scores were evaluated by a blinded observer. Besides LC-MS/MS and histology, other experiments were not performed in a blinded manner.

Reporting for specific materials, systems and methods

We require information from authors about some types of materials, experimental systems and methods used in many studies. Here, indicate whether each material, system or method listed is relevant to your study. If you are not sure if a list item applies to your research, read the appropriate section before selecting a response.

Materials & experimental systems

n/a	Involved in the study
☐	☒ Antibodies
☐	☒ Eukaryotic cell lines
☒	☐ Palaeontology and archaeology
☐	☒ Animals and other organisms
☐	☒ Human research participants
☒	☐ Clinical data
☒	☐ Dual use research of concern

Methods

n/a	Involved in the study
☒	☐ ChIP-seq
☐	☒ Flow cytometry
☒	☐ MRI-based neuroimaging

Antibodies

Antibodies used

FITC-conjugated anti-mouse CD45,Clone: BM8; Biolegend Cat# 103108, RRID: AB_312973
PerCP/Cyanine5.5-conjugated anti-mouse F4/80 antibody; Clone: BM8; Biolegend; Cat# 123128, RRID: AB_893484
PE/Cy7-conjugated anti-mouse Ly-6G/Ly-6C (GR-1); Clone: RB6-8C5; Biolegend; Cat# 108416, RRID: AB_313381

Validation

FITC-conjugated anti-mouse CD45; Biolegend Cat# 103108, RRID: AB_312973
PerCP/Cyanine5.5-conjugated anti-mouse F4/80 antibody; Biolegend; Cat# 123128, RRID: AB_893484
PE/Cy7-conjugated anti-mouse Ly-6G/Ly-6C (GR-1); Biolegend; Cat# 108416, RRID: AB_313381

All of the antibodies had been used in our previous studies for FACS analysis of immune cells (PMID: 33541845, 32594738, 32220957, 31760871, 31444155, 30803995, 29848663, and 29717038). These antibodies were also widely used by other laboratories.

Eukaryotic cell lines

Policy information about cell lines

Cell line source(s)

The MC38 cell line was a gift from Prof. Ajit Varki at UCSD, and was cultured in the lab of Dr. Zhang at the UMass-Amherst campus, and was used in our previous studies (PMID: 27933995, 28672272, and 30803995). Caco2 cells (HTB-37; https://www.atcc.org/products/htb-37) and HCT-116 cells (CCL-274; https://www.atcc.org/products/ccl-247) are from ATCC.

Authentication

The cells were not authenticated.

Mycoplasma contamination

The cells were not tested for mycoplasma contamination.

Commonly misidentified lines
(See ICLAC register)

No

Animals and other organisms

Policy information about studies involving animals; ARRIVE guidelines recommended for reporting animal research

Laboratory animals

C57BL/6 (male, 6-week age, wild type; conventional or germ-free) and Swiss Webster mice (female, 6-week age, conventional or germ-free).

Wild animals

No wild animals were used in the study.

Field-collected samples

No field collected samples were used in the study.

Ethics oversight

Animal experiments were conducted in accordance with protocols approved by the Institutional Animal Care and Use Committee of the University of Massachusetts (Amherst, MA) and Massachusetts Host-Microbiome Center at the Brigham and Women's Hospital (Boston, MA).

Note that full information on the approval of the study protocol must also be provided in the manuscript.

Human research participants

Policy information about studies involving human research participants

Population characteristics

Human urine and stool samples from a previous study (see publication PMID: 27303746) were kindly provided by Dr. Julie Parsonnet as a gift, then the samples were analyzed by LC-MS/MS.

Recruitment

The information of recruitment was described in the previous publication (PMID: 27303746): healthy volunteers were recruited from July 2011 to March 2013 using flyers, talks, and informal discussions. Inclusion criteria includes: (1) age greater than 18 years, and (2) willingness to use study-assigned HPCPs (toothpaste, hard and liquid soap, dish soap) for 8

	months. Exclusion criteria includes: (1) recent use of antibiotics, gastrointestinal illness, (2) travel to a developing country within the prior 3 months, (3) pregnancy, (4) unwillingness to change personal hygiene products, and (5) high likelihood of noncompliance.
Ethics oversight	The original human study (see publication PMID: 27303746) was approved by the Institutional Review Board of Stanford University (ClinicalTrials.gov identifier NCT01509976).

Note that full information on the approval of the study protocol must also be provided in the manuscript.

Flow Cytometry

Plots

Confirm that:

- [x] The axis labels state the marker and fluorochrome used (e.g. CD4-FITC).
- [x] The axis scales are clearly visible. Include numbers along axes only for bottom left plot of group (a 'group' is an analysis of identical markers).
- [x] All plots are contour plots with outliers or pseudocolor plots.
- [x] A numerical value for number of cells or percentage (with statistics) is provided.

Methodology

Sample preparation	Distal colon tissues were dissected, washed with cold PBS, and digested with Hank's balanced salt solution (HBSS, Lonza) supplemented with 1 mM dithiothreitol (DTT) and 5mM EDTA at 4°C (colon epidermal cells).
Instrument	BD LSR FortessaTMcell analyzer (Becton Dickinson, Franklin Lakes, NJ)
Software	FlowJo software (FlowJo, LLC).
Cell population abundance	collected: 10000 cells per sample<br>showed: 1000 cells per sample
Gating strategy	Cells were stained with Zombie VioletTM dye (Zombie VioletTM Fixable Viability Kit; BioLegend) according to the manufacturer's instructions to exclude dead cells. Gating and cell identification strategies are as follows: briefly, cell doublets and clumps were eliminated using FSC-A gating and debris was eliminated using FSC-A vs SSC-A. Dead cells were gated out using Zombie Violet™ dye.

- [x] Tick this box to confirm that a figure exemplifying the gating strategy is provided in the Supplementary Information.
